# Detecting experimental techniques and selecting relevant documents for protein-protein interactions from biomedical literature

**DOI:** 10.1186/1471-2105-12-S8-S11

**Published:** 2011-10-03

**Authors:** Xinglong Wang, Rafal Rak, Angelo Restificar, Chikashi Nobata, CJ Rupp, Riza Theresa B Batista-Navarro, Raheel Nawaz, Sophia Ananiadou

**Affiliations:** 1 National Centre for Text Mining and School of Computer Science, University of Manchester, Manchester, UK

## Abstract

**Background:**

The selection of relevant articles for curation, and linking those articles to experimental techniques confirming the findings became one of the primary subjects of the recent BioCreative III contest. The contest’s Protein-Protein Interaction (PPI) task consisted of two sub-tasks: Article Classification Task (ACT) and Interaction Method Task (IMT). ACT aimed to automatically select relevant documents for PPI curation, whereas the goal of IMT was to recognise the methods used in experiments for identifying the interactions in full-text articles.

**Results:**

We proposed and compared several classification-based methods for both tasks, employing rich contextual features as well as features extracted from external knowledge sources. For IMT, a new method that classifies pair-wise relations between every text phrase and candidate interaction method obtained promising results with an F1 score of 64.49%, as tested on the task’s development dataset. We also explored ways to combine this new approach and more conventional, multi-label document classification methods. For ACT, our classifiers exploited automatically detected named entities and other linguistic information. The evaluation results on the BioCreative III PPI test datasets showed that our systems were very competitive: one of our IMT methods yielded the best performance among all participants, as measured by F1 score, Matthew’s Correlation Coefficient and AUC iP/R; whereas for ACT, our best classifier was ranked second as measured by AUC iP/R, and also competitive according to other metrics.

**Conclusions:**

Our novel approach that converts the multi-class, multi-label classification problem to a binary classification problem showed much promise in IMT. Nevertheless, on the test dataset the best performance was achieved by taking the union of the output of this method and that of a multi-class, multi-label document classifier, which indicates that the two types of systems complement each other in terms of recall. For ACT, our system exploited a rich set of features and also obtained encouraging results. We examined the features with respect to their contributions to the classification results, and concluded that contextual words surrounding named entities, as well as the MeSH headings associated with the documents were among the main contributors to the performance.

## Background

In the domains of Biomedicine and Genomics, scientific discoveries and empirical knowledge are often buried in the vast amount of research publications, doctors’ notes, and other forms of text. This poses a challenge to scientists and medical practioners in accurately and efficiently locating specific pieces of information. Addressing this need, text mining technologies have shown promise in helping to accelerate the organisation and curation of biomedical literature [1-3].

Devoted to the evaluation of biomedical IE systems, BioCreative challenges (Critical Assessment of Information Extraction in Biology) [4,5] is a series of events that brings together computational linguists, computer scientists, and biomedical researchers to develop, test and exchange text mining ideas in the context of biomedical literature curation. The extraction of protein protein interactions (PPIs) has been one of the main topics in the BioCreative series of workshops.

Although techniques for mining PPIs have improved in recent years [4-6], the need for associating evidence and attributes to PPIs so they could easily be processed and linked together, either by human users or automated systems, has yet to be sufficiently addressed. For example, experimental techniques applied for discovering PPIs, such as *yeast two-hybrid screening* and *anti tag coimmunoprecipitation*, are important for understanding the findings and for validating and reproducing the results. BioCreative II’s Interaction Method Subtask (IMS) [4] was designed to tackle this challenge. Two teams [7,8] participated in the task and the best F1 score was reported at a level of 45%, obtained by matching the document text against a set of variants of the interaction method terms in the ontology provided.

Along similar goals established in BioCreative II, the BioCreative III Interaction Method Task (IMT) is particularly directed towards the development of automated systems that detect the techniques used in experiments to confirm a given PPI from full-text research articles. The permitted set of interaction methods comes from the PSI-MI ontology [9] and consists of a subset of 115 definitions of experimental techniques. In this task each article may be associated with zero or more methods. Therefore, the task can be cast as a multi-class, multi-label document classification problem. The interaction methods from PMI-MI are represented by their unique identifiers, henceforth referred to as MI IDs.

IMT bears some resemblance to an entity normalisation task (e.g., the gene normalisation tasks in the BioCreative challenges [5,10,11]), in that for each given document, if the terms describing interaction methods were recognised and grounded to MI IDs, the task would be solved. Therefore, we also approached the task as term normalisation, using string similarity measures, and then training a model to filter out false positives.

The aim of the Article Classification Task (ACT) is to categorise documents as being relevant or irrelevant to PPI curation. ACT addresses a time-consuming but essential task in a typical manual curation work-flow: a curator spends a significant amount of time scanning through a paper to establish whether the document in question contains curatable PPIs. According to the task specification, only documents reporting PPIs are considered relevant, while those describing interactions between genes or other non-protein biological entities are not.

Tasks similar to ACT have attracted much research attention, most notably through Interaction Articles Subtask (IAS) in BioCreative II [4] and ACT in BioCreative II.5 [5]. Both abstracts and full-text papers were provided in BioCreative III’s ACT, but only information from the abstracts were considered during evaluation. We cast ACT as a binary document classification task, and our strategy was to exploit a rich set of features including linguistic information and named entities that were automatically annotated using text mining systems.

## Results and discussion

### Data

The organisers of the challenge provided three datasets for each task, referred to as *training*, *development*, and *test*. The former two datasets were intended to be used in the process of developing the systems by the tasks’ participants. Once the systems were ready, the participants were asked to submit the results on the test dataset, for which the annotation was (initially) unknown. Table 1 shows the distribution of articles in each dataset for each task. We used the PSI-MI ontology [9] provided by the European Bioinformatics Institute (EBI) for grounding experimental techniques to MI IDs.

**Table 1:** Distribution of articles in the training, development, and test datasets

Task	Training	Development	Test	Scope
IMT	2,035	587	305	full-text
ACT	2,280	4,000	6,000	abstract and full-text

Distribution of articles in the training, development, and test datasets provided by the BioCreative III organisers for each of the PPI tasks.

### Detecting interaction methods

We devised three distinct systems for IMT, two of which followed the more common multi-class, multi-label document classification framework, and the other one used a binary model to classify pairs of method names and text phrases. We experimented with two machine-learning paradigms, namely Logistic Regression (LR) and Support Vector Machines (SVM). SVM was chosen because it has been shown to be superior to other commonly used machine learning methods for classification [12]. Specifically, SVM demonstrated good performance on a range of tasks in biomedical text mining, including but not limited to, document classification [13], named entity recognition [14], named entity disambiguation [15], relation extraction [6] and bio-event extraction [16].

LR is one of the most widely-used probabilistic modeling techniques in the field of natural language processing and has also been shown to be effective in handling large-scale classification problems [17,18]. The applications range from simple classification tasks such as text categorisation [19] to more complex structured prediction tasks such as part-of-speech (POS) tagging [20] and named entity recognition [21]. LR models produce probabilistic output, which allows the information on the confidence of the decision to be used by subsequent components in the text processing pipeline, and this is an advantage over other discriminative machine learning models such as SVM.

In our experiments, both SVM and LR were used in the multi-class, multi-label classification scenario, whereas the binary model utilised SVM only. We refer to the three models as *m-LR*, *m-SVM*, and *b-SVM*, respectively, and we elaborate on these systems in Section Methods.

We performed cross-validation experiments on the training dataset, based on which the best models were selected and further tested on the development dataset. For each document, we also took the union and the intersection of sets of MI IDs obtained from the aforementioned approaches. The results are shown in Table 2. As expected, the union of results improved recall and the intersection improved precision. Based on the results, we chose to submit the systems marked with asterisks (*), for which we trained models on the combined training and development datasets, and then applied the models on the test dataset.

**Table 2:** IMT results on the development dataset

System	Precision	Recall	F1 score	AUC iP/R
m-LR	41.36	53.81	46.37	22.85
m-SVM	72.12	51.31	59.96	39.05
b-SVM (*)	68.35	61.05	**64.49**	42.02
union(m-SVM,b-SVM) (*)	65.62	63.11	64.33	**44.98**
union(m-LR,b-SVM)	42.33	**64.36**	50.73	27.76
union(m-LR,m-SVM)	41.39	54.01	46.46	**22.94**
intersect(m-LR,b-SVM) (*)	75.24	54.96	63.52	44.02
intersect(m-LR,m-SVM,b-SVM) (*)	**78.22**	50.17	61.13	40.92

Macro-averaged results on the IMT development dataset with 10 best models selected by cross-validation on the training data (%). m-LR – multi-label Logistic Regression; m-SVM – multi-label Support Vector Machines; b-SVM – binary Support Vector Machines. Asterisks (*) denote systems that were submitted to the challenge.

Table 3 shows the results on the test dataset. To our surprise, the scores obtained on the test dataset were inconsistent with those on the development dataset. For example, m-LR and m-SVM outperformed b-SVM by nearly 5% points in F1 score. Another observation is that the union systems that join individual system outputs have performed consistently well. In particular, combining results from m-LR or m-SVM with those from b-SVM yielded the best results.

**Table 3:** IMT results on the test dataset

System	Precision	Recall	F1 score	AUC iP/R
m-LR	58.37	55.80	57.06	34.96
m-SVM	62.33	48.94	54.83	33.18
b-SVM (*)	52.56	52.45	52.50	28.45
union(m-SVM,b-SVM) (*)	53.21	59.61	56.23	35.85
union(m-LR,b-SVM)	52.51	**64.67**	**57.96**	**39.06**
union(m-LR,m-SVM)	57.43	56.18	56.80	35.26
intersect(m-LR,b-SVM) (*)	64.06	44.01	52.17	29.47
intersect(m-LR,m-SVM,b-SVM) (*)	**64.86**	44.42	52.73	30.52

Results on the IMT test dataset (%). The models were trained on the combined training and development datasets. m-LR – multi-label Logistic Regression; m-SVM – multi-label Support Vector Machines; b-SVM – binary Support Vector Machines. Asterisks (*) denote systems that were submitted to the challenge.

**Figure 1:**
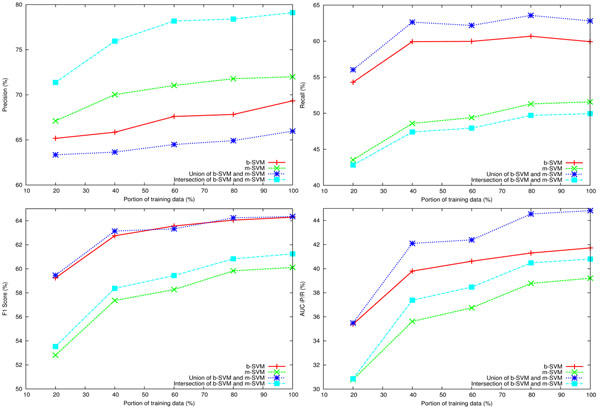
**Learning curves of the IMT systems**. Figure 1 shows learning curves of the following IMT systems: b-SVM, m-SVM, the union and the intersection of the output of b-SVM and m-SVM, as measured by precision, recall, F1 score and AUC iP/R. Each system was trained using increasing amounts of the data, i.e., 20%, 40%, 60%, 80% an 100% of the training dataset, and then tested on the development set.

To investigate whether there was an overfitting problem, we plotted learning curves for the better performing systems as tested on the development data: m-SVM, b-SVM, and the union and the intersection of the two systems. Figure 1 consists of 4 graphs, each plotting learning curves of the 4 systems using the following evaluation metrics: precision, recall, F1 score and AUC iP/R. Each system was trained on increasing amounts of data, randomly taking 20%, 40%, 60%, 80%, and finally the entire set of the training data; whereas the performance was recorded on the development dataset. As shown in the figure, the system that takes the union of the results obtained from b-SVM and m-SVM excels in most of the cases. Also, according to all measures except recall, performances went up as the size of training data increased, indicating overfitting did not occur on the training and development datasets.

**Figure 2:**
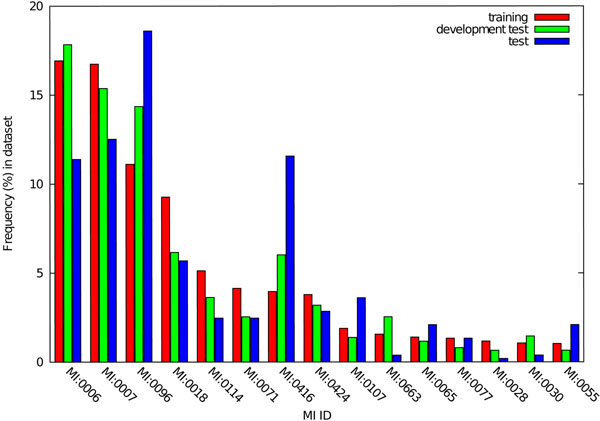
**Comparison of MI ID distributions in IMT training, development and test datasets.** Figure 2 plotted histograms showing distribution of frequencies of MI IDs in the training, development a test datasets, respectively.

However, the performance decreased when the models were trained on the combined training and development datasets, and tested on the test dataset. Alongside the above analysis, it suggests that the distributions of MI IDs are likely to be similar between the training and development datasets, but different between the training and test datasets. Figure 2 shows the histograms of the 15 most frequent MI IDs as found in the training dataset, together with their corresponding frequencies in the development and test datasets. The figure reveals that, in the training and development datasets, the 4 most frequently occurring MI IDs and their order are identical, which in both datasets account for over 50% of all occurrences of MI IDs. In the test dataset, on the other hand, the frequencies of these IDs are distributed differently. As a consequence, it was likely that b-SVM was overfitted to the training and development datasets. Indeed, the different class distribution affects b-SVM more than m-SVM and m-LR, because some features used by b-SVM are tuned to the distributions of the MI IDs in the training data, e.g., the features that map the frequent MI IDs and their MeSH equivalents. In summary, under the current experiment settings, the multi-classification approaches showed better adaptability to a diverse range of datasets, while b-SVM needs to minimise its dependence on training data by feature engineering or algorithm tuning, in order to achieve better portability.

To gain insight on how the systems could possibly be improved in the future, we performed manual error analysis following the steps listed below:

1. Based on the performance results on the development set, a set of most frequently occurring MI IDs for which all systems produced incorrect predictions was extracted. This step showed that all systems performed poorly for the following MI IDs: MI:0028, MI:0030, MI:0071, MI:0416 and MI:0663.

2. Focusing on, but not limiting to, the list of MI IDs from the first step, we randomly selected 20 document - MI ID pairs from the list of incorrect predictions (accounting for both false negatives and false positives). We analysed the corresponding full-text articles and gold standard annotations, manually looking for information which could have been missed (in the case of false negatives) or wrongly interpreted (in the case of false positives) by our systems.

Our findings showed that the errors can be attributed to: 1) more challenging aspects of the problem which our systems have not addressed yet; and 2) some inconsistent annotations in the training and development sets.

Most of the false positives resulted from recognising mentions of method names which occurred in the text but not in the description of how a protein interaction was established. For example, some authors, in describing their failed attempts to detect protein interactions, mention the methods they have used. This resulted in a number of false positives, as the task requires that only methods which support an established interaction are to be considered. This implies that it is necessary for a system to consider only mentions of methods in the context of an established protein interactions which, in turn, suggests that perhaps protein interactions need to be extracted beforehand.

On the other hand, several inconsistent annotations were found both in the training and development datasets. For example, the documents with PMIDs 16339921, 16456538, 16467851 and 16478466 which describe “confocal microscopy” were not annotated with MI:0663 (*confocal microscopy*). Having been trained on such data, the systems may have failed to learn the association between the concept and the documents which describe it. This possibly resulted in the poor recall on the development set with respect to a few MI IDs such as MI:0663.

In addition, while the annotation guidelines seem to specify that the most specific possible MI concept should be assigned to a document, we observed that it was not always the case. For instance, the document PMID 19804757 had been annotated with only MI:0004 (*affinity chromatography*); a review of the article, however, reveals that the methodology used in detecting the protein interactions is “anti tag coimmunoprecipitation” which is an indirect subtype of *affinity chromatography* in the PSI-MI ontology. We also found that some documents contain incorrect annotations. For example, the document PMID 19411070 was annotated with MI:0416 (*fluorescence microscopy*) even though this method was not mentioned in the main text, but only in the supplementary material which was not included in the provided data set. In conclusion, although we were unable to systematically measure how much the annotation errors affected the systems’ results, we can speculate that an improvement to annotation consistency would result in better and more reliable models.

### Selecting articles relevant to PPI curation

For ACT, we experimented with both SVM and LR, as well as different types of MeSH information used as features. The first type of MeSH feature was based purely on the unique identifier of a MeSH term (MeSH ID), whereas the second one was based on a more elaborate, tree-structure representation. In the course of experiments we eventually focused on three distinct systems: two based on SVM and one based on LR. The detailed description of the systems is provided in Section Methods.

The performance of the three systems tested on the test dataset is given in Table 4; while Table 5 shows 10-fold cross-validation results on the combined training and development datasets.

**Table 4:** ACT results on the test dataset

System	F1 score	Specificity	Sensitivity	Accuracy	Matthews Coef	AUC iP/R
SVM_MeSH ID_	57.44	**96.03**	49.23	**88.93**	52.237	49.26
SVM_MeSH Tree_	59.01	94.97	53.63	88.70	52.890	51.65
LR_MeSH ID_	**59.64**	93.93	**56.92**	88.32	**52.914**	**65.24**

Results on the ACT test dataset (%). SVM_MeSH ID_ – SVM with MeSH identifiers; SVM_MeSH Tree_ – SVM with MeSH tree structure; LR – LR with MeSH identifiers.

**Table 5:** ACT 10-fold cross-validation results on the training and development datasets

System	F1 score	Specificity	Sensitivity	Accuracy	Matthews Coef	AUC iP/R
SVM_MeSH ID_	75.87	94.26	69.70	87.13	67.68	75.11
SVM_MeSH Tree_	**77.01**	**94.48**	71.08	**87.69**	**69.14**	76.22
LR_MeSH ID_	76.78	93.49	**72.23**	87.33	68.37	**82.89**
SVM_MeSH ID&Tree_	76.90	**94.48**	70.91	87.64	69.01	76.20

The 10-fold cross-validation results for ACT on the training and development datasets (%). SVM_MeSH ID_ – SVM with MeSH identifiers; SVM_MeSH Tree_ – SVM with MeSH tree structure; LR – LR with MeSH identifiers; SVM_MeSH ID&Tree_ – SVM with both MeSH identifiers and tree structure.

The cross validation experiment results formed the basis for choosing models for testing, and the experiment was carried out on the combined set because the number of training instances for ACT was much smaller than the corresponding development set (2,280 vs. 4,000 abstracts), and joining the two datasets for cross validation resulted in a larger amount of training data, leading to better classification models. Table 5 shows that the LR system obtained better results as measured by AUC iP/R than the SVM ones, which indicates that LR produced better ranking than SVM. In addition, comparing tables 4 and 5, the results are lower on the test dataset than on the combined, cross-validated training and development datasets. One reason may be the difference in the ratios of the amounts of training and test datasets used: in the 10-fold cross-validation experiments (See Table 5), the size of the training data was 9 times more than that of the test data; whereas in the other set of experiments (See Table 4), the two datasets were of similar size (6,280 vs. 6,000 abstracts). As using a larger amount of test data tends to produce more stably estimated performance, Table 4 reveals that the models prepared using the combined training and development datasets are still not satisfactory in providing good coverage.

Table 5 also provides evidence on which MeSH information is more useful in classifying documents relevant to protein interactions. It appears that the system employing MeSH tree structure (i.e., SVM_MeSH Tree_) yielded better results than the one using MeSH IDs. This is due to the fact that MeSH tree-structure representation had the capability to generalise over MeSH categories, and was therefore able to alleviate data sparseness problem of MeSH. When both MeSH IDs and MeSH structure were used (SVM_MeSH ID&Tree_in Table 5), the results were slightly worse than those with MeSH structure only. Table 6 shows the evaluation results when only the training dataset was employed for training and the development set for testing. As expected, the evaluation results were much worse than the cross-validation results shown in Table 5.

**Table 6:** ACT results on the development dataset

System	F1 score	Specificity	Sensitivity	Accuracy	Matthews Coef	AUC iP/R
SVM_MeSH ID_	14.05	**96.62**	8.80	**81.65**	10.05	20.75
SVM_MeSH Tree_	15.68	96.11	10.12	81.45	**10.80**	**21.04**
LR_MeSH ID_	**27.01**	61.09	**45.16**	58.38	4.80	19.16

Results on the ACT development dataset with models trained on the training dataset (%). SVM_MeSH ID_ – SVM with MeSH identifiers; SVM_MeSH Tree_ – SVM with MeSH tree structure; LR – LR with MeSH identifiers.

**Figure 3:**
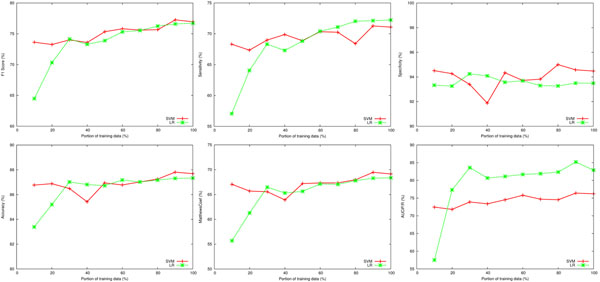
**Learning curves of the ACT systems.** Figure 3 shows how the size of training data affected ACT performance. We gradually increased the training size from 10% to 90%. The training data at each percentage point was randomly selected, and then 10-fold cross validations was performed and results plotted.

Similarly to IMT, we also conducted an experiment to see how the size of training data affects the models, the results of which are shown in Figure 3. The size of the data was gradually increased from 10% to 100%, where data at each percentage mark was randomly selected from the combined training and development datasets. We then performed 10-fold cross validations on the selected data. The figure shows that the performance of the SVM system was rather indifferent to the size of data used to train the model. The LR system requires more training data as the curve is steep up to the 30% mark in F1 score, and then the slope gradually decreases as the size of data increases.

## Conclusions

We compared several approaches to the BioCreative III IMT and ACT tasks. For IMT, we proposed a new method that first searches for candidate interaction method text strings in documents, and then classifies pair-wise relations between the candidates and their matching interaction method names, as defined in PSI-MI. This method utilises a rich set of features extracted from the candidates’ surrounding context, together with the definitions and synonyms in PSI-MI. Evaluation results on the development dataset show that, overall, this method is promising and outperforms the more conventional multi-label document classification using the “one-vs-all” strategy. However, its superiority was not confirmed on the test dataset, and the variance indicates that the model may be overfitted to the training and development data. We also tested simple ensemble systems using heuristic rules of union and intersection to combine methods, and achieved very good overall performance on both test datasets, which provided evidence that the systems complement each other.

For ACT, we tested LR and SVM classifiers exploiting a rich set of features obtained by linguistic processing and automatic recognition of a wide range of biomedical named entities. A series of feature-knockout experiments (See Section Methods) showed that the discriminative power is drawn mainly from contextual words surrounding automatically recognized named entities, as well as MeSH.

## Methods

### Document pre-processing

The organisers of the challenge provided the full-text articles in various formats, of which we used the PDF-converted plain text format. Unfortunately, the quality of the text was not satisfactory, but we did not find a quick solution to address this issue. Nevertheless, we normalised typographic ligatures, Unicode punctuation, such as different white spaces, dashes, single and double quotes, and also removed control characters. By contrast, the ACT documents (abstracts only) were of good quality, and therefore we did not apply the above text-cleaning procedures.

Both the IMT and ACT documents were pre-processed using a number of linguistic processors [2], including tokenisation, lemmatisation, part-of-speech tagging and chunking, and were also processed with a named entity recogniser (NER). We used an in-house NER, which is also employed in our semantic search engine Kleio [22], based on the method described in [23]. The NER consists of two components: the first finds entity candidates by searching a dictionary; and if a manually annotated corpus is available, the second component trains a conditional-random-fields model on the JNLPBA dataset [24], which in turn is used to tag unseen text. We applied both components for annotating genes and proteins, and only the first for annotating the following types of entities: metabolites, organs, drugs, bacteria, diseases, symptoms, diagnostic/therapeutic procedures and phenomena. Please refer to [25] for more details regarding the NER. Text descriptions of the methods from the PSI-MI ontology were pre-processed using the same linguistic pipeline, but without named entity recognition. Together with MI names and synonyms, the key words in the text descriptions are good indicators of the occurrence of their corresponding MI IDs, and therefore they were used as features in the b-SVM method, as described later in this section.

Additionally, for each IMT and ACT document, we retrieved its MeSH entries and other associated information from the MeSH ontology [26]. The MeSH information of interest includes terms’ names and identifiers, in both their atomic and hierarchically ordered form (i.e., tree ID), with the latter more closely representing the underlying structure of MeSH ontology. This information was used as a feature in training machine-learning models.

For IMT, we also manually constructed a mapping from the 10 most frequent MI IDs (as found in the training data), to their corresponding MeSH terms. MeSH and the PSI-MI ontology differ significantly in terms of scope, generality and organisation. However there is an overlap between the two hierarchies. For example, MeSH has a branch (E05: *Investigative Techniques*), which is similar to our branch of interest (MI001: *interaction detection method*) in the PSI-MI hierarchy. Having observed this overlap, we decided to use the relevant information from MeSH as an additional source for the IMT task.

Mapping between PSI-MI and MeSH was done manually. We first identified the most frequent PSI-MI terms in the training dataset, and then tried to find their equivalent terms in MeSH. We used the MeSH Browser [26] for searching and browsing through the MeSH ontology, and we followed a simple sequence of steps to find a MeSH equivalent for a given PSI-MI term:

1. Search the MeSH ontology for an exact match for the given MI term. If a match is not found, move to step 2.

2. For each given synonym of the MI term, try to find an exact match in the MeSH ontology. If no matches are found, move to step 3

3. Search the MeSH ontology for an approximate match for the given MI term. If a match is not found, move to step 4. We only considered two types of approximate matches:

• Partial Matches: where one term contains fragments of the other term. For example, the MeSH term *Molecular Sieve Chromatography* (E05.196.181.400.250 ) contains two fragments of the PSI-MI term *Molecular Sieving* (MI:0071).

• Hyphenated/Unhyphenated Variants: where a hyphenated or unhyphenated variant of the PSI-MI term is present in the MeSH ontology. For example, the PSI-MI term *coimmunoprecipitation* (MI:0019) has a hyphenated variant *Co-Immunoprecipitation* (E05.196.150.639 ) present in the MeSH ontology.

4. For each given synonym of the MI term, try to find an approximate match in the MeSH ontology.

In cases where we failed to find a match for the given MI term, we tried to find a match for the parent term. Table 7 shows the manually built mapping.

**Table 7:** Mapping between MI IDs and MeSH terms

Rank	MI ID	MI name	MeSH ID	MeSH term
1	MI:0007	anti tag coimmunoprecipitation	E05.196.150.639	Co-Immunoprecipitation
2	MI:0006	anti bait coimmunoprecipitation	E05.196.150.639	Co-Immunoprecipitation
3	MI:0096	pull down	E05.196.181.400.170	Affinity Chromatography
4	MI:0018	two hybrid	E05.393.220.870	Two-hybrid System Techniques
5	MI:0114	X-ray crystallography	E05.196.309.742.225	X-Ray Crystallography
6	MI:0071	Molecular Sieving	E05.196.181.400.250	Molecular Sieve Chromatography
7	MI:0416	Fluorescence Microscopy	E01.370.350.515.458	Fluorescence Microscopy
8	MI:0424	Protein Kinase Assay	E05.196.630.570.700	Protein Array Analysis
9	MI:0107	Surface Plasmon Resonance	E05.196.890	Surface Plasmon Resonance
10	MI:0663	Confocal Microscopy	E01.370.350.515.395	Confocal Microscopy

The manually constructed mapping between interaction methods from the PSI-MI ontology and MeSH terms, ranked by occurrence frequency in the training data

### Approaches to interaction method detection

IMT is a multi-class, multi-label classification problem and all three of our systems, i.e., m-LR, m-SVM, and b-SVM, approach IMT by training binary classifiers. m-LR and m-SVM use different machine learning techniques (i.e., LR and SVM) to fit the models but use the same set of features. Both m-LR and m-SVM use the one-vs-all scheme with thresholding, where models that output a value greater than a threshold determine the labels to be assigned to a given document. b-SVM also uses SVM to fit the models, but compared to m-LR and m-SVM, it exploits a different feature set, and more importantly, poses the multi-class, multi-label classification problem differently: instead of fitting multiple binary classification models as m-LR and m-SVM do, it only fits one binary classification model from the training data.

#### Classifying pairs of text chunks and method names for IMT

The b-SVM system extracts every text chunk in a full-text document and collects the chunks that are approximately similar to an interaction method name or synonym in the PSI-MI ontology, where the strength of similarity is determined by a string similarity measure. This technique was borrowed from the name matching tasks [27] and was also adopted by some teams [28,29] participating in the previous BioCreative gene normalisation tasks. The intuition was that if a text chunk looks similar to an PSI-MI name (e.g., “pull down” vs. “pull-down”), they are likely to refer to the same concept. In our work, the text chunks were noun phrases (NP) and verb phrases (VP) tagged by an in-house text chunker [2]. In total, nearly 1 million chunks were collected from the 2,035 full-text articles in the training dataset, which gave an average of 486 chunks per document. Each chunk was then coupled with a PSI-MI name or its synonym to form a pair. The names and synonyms were extracted from the list of allowed MI IDs supplied by the task organisers.

A number of string similarity measures could be used for this task. We chose SoftTFIDF [27] based on our previous work and experience [28,29]. SoftTFIDF is similar to the widely known TFIDF, except that when calculating term frequency and inverse document frequency, similar tokens are considered as well as the tokens in the two strings. The secondary similarity function we used was JaroWinkler [30], with threshold 0.85. This way, if the text chunk and PSI-MI name in a pair had a SoftTFIDF similarity score above 0.50, the pair was kept for further classification, where both thresholds were determined by cross-validation tests using the training data. The classification step was necessary because the similarity of two strings does not guarantee they refer to the same concept. For example, strings “fax” and “fac” (MI:0054) are similar according to SoftTFIDF but they refer to separate concepts in this context. Consequently, such false positives need to be filtered out on the basis of the surrounding context of the text chunk and the properties of the MI ID.

After such pairs were collected, each pair was classified as positive, indicating that the text chunk in question entails the corresponding interaction method, or negative, otherwise. All MI IDs appearing in the *positive* pairs were then assigned to the document. In more detail, suppose a document contains NP and VP chunks; we compare each chunk to every name in PSI-MI, and gather all pairs whose SoftTFIDF scores are above 0.5. For example, if a pair consists of an NP chunk, “anti-His tag antibodies”, and a method name “anti tag coimmunoprecipitation” (MI:0007), then according to SoftTFIDF, their similarity is 0.834, surpassing the defined threshold (i.e., 0.5). The trained model is then applied to the pair, and if it is classified as positive, MI:0007 would be assigned to the document in question.

This way, a multi-label document classification problem is approached by training just one binary model, simplifying the machine learning task; and if we carefully choose features that depict the relation between a chunk and an MI ID, by, for example, looking at how much the chunk’s surrounding context overlaps the description of the ID in PSI-MI, the actual content of the chunk and the ID become less important. In other words, the performance of classifying such pairs is less dependent on the amount of training data available for the MI ID in question. Hence, the approach addressed the problem faced by multi-label document classification where many MI IDs do not have sufficient data to train a good model. However, if features are too dependent on specific MI IDs, the model would be biased to the distribution of classes in the training data.

We used the SVM*^per f^* classifier (with the linear kernel) [31,32] in b-SVM. More specifically, we define a candidate pair to be classified as *p* = {*c*, *n*}, where *c* is a text chunk and *n* is an MI name that matches *c*. We also define *id* as *n*’s corresponding MI identifier and the document containing *c* as *D.* In training, given a labelled document *D*, a training instance derived from *D* is a positive example only if the method name (i.e., *n*) in *p* = {*c*, *n*} is one of the gold-standard annotations of *D.* Any other training instance derived from *D* is considered as a negative example. During inference, given a document *D'*, b-SVM outputs *id* corresponding to *n* in *p* = {*c*, *n*} as a label for *D'* only in the case that b-SVM predicts *p* as a positive instance derived from *D'.* The features used for training and inference are listed below.

**Local context** includes contextual words within a defined window surrounding *c*. We chose two window sizes: 10 and 50 with the former additionally accompanied by position information.

**Local NER context** is the named entities adjacent to *c*. We took 5 on each side and both the type (e.g., protein) and text of the entities were used. All types of entities supported by our NER system were included, namely, protein, gene, metabolite, organ, drug, bacteria, disease, symptom, diagnostic/therapeutic procedure and phenomenon.

**MI synonym match** We searched the local context (window size 20) of *c* and the global context of *D* (i.e., all tokens in *D*) for the names and synonyms linked to *id* in PSI-MI. The number of matches in both cases were used as features.

**MI definition match** In addition to names and synonyms, key words in the definition associated with *id* may be useful. We ranked the tokens in each definition according to their TFIDF scores so that the tokens at the top of the rank were more likely to relate to *id*. Given this rank, we searched the local context (window size 20) of *c* and the global context of *D* for tokens in *id*’s definition, and then used the TFIDF rank linked to each definition token as a binary feature.

**Section title** Based on the assumption that method names are more likely to be mentioned in some sections (e.g., “Materials and Methods”) than others, we searched the text for the commonly used section names in biomedical articles, such as “introduction” and “results and discussion”, tagged them as section titles and used these as features.

**MeSH terms** A feature indicating whether *D* is annotated with a MeSH term that matches one of the top 10 frequently occurring MI IDs (based on their counts in the training data), using the manually built mapping described above.

**Other features** These include the text strings of *c* and *id*, and the string similarity score between *c* and *n*. Note that all contextual words were lemmatised and “stop words” (e.g., functional words and words consisting of only digits) were removed. We tuned the *C*-value of SVM by cross-validation on the training set and achieved the best F1 score with the values 16 or 32. The final model was trained on the training and development datasets.

#### Multi-label document classification for IMT

We also approached IMT as multi-label document classification, using an ensemble of binary classifiers produced for each class (i.e., MI ID). We trained the binary classifiers using the *one-vs-all* strategy, where each model was trained on all instances, and positive instances were those that belong to a class for which the model was being built. Each document was scored by all the models, and if the score for a particular document was greater than the corresponding threshold for a particular model, the label associated with that model was assigned to that document.

The features used were different from those in b-SVM and included the following: type and text of named entities, words surrounding the entities (window size 10 with position), the title of the section in which the entities occur; as well as an indication whether there are matches between the word unigrams and character n-grams (*n* = {2,3,4}) from the PSI-MI definition and synonyms, and those in the documents. The motivation for using n-grams was to allow the PSI-MI definition to use, as features, words that may be spelled slightly differently in the document. By chopping the words in the definition into smaller chunks and checking the presence of these n-grams in the document would also provide us with some measure of distance. The feature is set to 1 only if the character n-gram is present in both the PSI-MI definition and synonyms *and* the document. The probability that two instances within the same class have the same features switched on, is proportional to how similar the distributions of document words are in the two documents that correspond to those two instances. Based on this set of features, we tested two machine learning algorithms: LR and SVM (referred to as m-LR and m-SVM respectively).

**Logistic Regression** We used models trained via *L*_2_-regularised LR [17,18] from instance vectors constructed using the features described above. In total, 85 LR models were constructed, one for each interaction method for which at least one instance in the training set was found. In order to assess how the LR models would generalize on unseen data, both the training data and development data provided by the organisers were used in a 10-fold cross-validation experiment. In this experiment, we have set aside the development data as a test set and decided to use the training data to build our models and also determine the threshold value.

We first randomly divided the data into 10-folds, and then performed 10 runs using the training data to build models and separately used the development data for testing. For each run, we used 9 folds to train the LR model *LR_j_*, *j* ∈ [1, 85], and the remaining fold to determine the threshold for *LR_j_*. Thus for each run, we trained 85 LR models. During training, a document *D* corresponds to one training instance. For a specific interaction method *MI_j_*, *j* ∈ [1, 85], we associate a corresponding LR model *LR_j_*. *D* is a positive example for *LR_j_* if it has been assigned a label *MI_j_* in the training data, otherwise *D* is a negative example for *LR_j_*. We performed 10 experiment runs, training a total of 850 LR models and averaged the results of the evaluation on the development dataset, as shown in Table 2. To construct the final model for the official test data, we used the training data to train the 85 LR models and the development set to determine the thresholds for each model, subject to the constraint that each threshold has a minimum value of 0.10.

**SVM** The implementation of SVM used was SVM*^per f^* with the linear kernel. The parameter *C*-value was tuned using 10-fold cross-validation on the training set in a fashion similar to the LR classification. Since the applied range of parameter values produced the same micro-average F1 score, we arbitrarily chose *C* = 1 for the final model.

**Thresholding Strategies** It has been argued that simple thresholding on scores obtained from classifiers does not always yield the best performance in multi-label classification. Several thresholding strategies have been proposed, which can be categorised into rank-based thresholding, class proportion-based assignment, and score-based local optimisation [33]. The ranked-based strategy involves sorting classes for each document according to their scores and choosing the top scoring classes, which will constitute the classification decision for the document. The class proportion-based assignment, on the other hand, is class-oriented and chooses a portion of documents with the highest scores. The scored-based optimisation assigns a class to a document based purely on the score between the two and a class-specific threshold. A popular variation of the score-based optimisation involves parameterising one global threshold for all the classes.

Since the ranked-based strategy selects only a fixed set of classes and the class proportion-based assignment is not suitable for an on-line classification, we chose to experiment with the score-based optimisation strategy. We compared the performance classifying documents using local (class-fitted) thresholds and global thresholds. The thresholds were tuned using 10-fold cross-validation on the training set, whereas the evaluation was performed on the development set. In the case of SVM, the classification with local thresholds resulted in an inferior (albeit marginally) micro-average F1 score to the classification with the global nominal (zero) threshold. That could indicate that the local thresholds over-fitted the training set, which was less likely with the global thresholding.

It is important to note that local thresholding driven to optimise F1 score for individual classes outperformed the global thresholding in terms of per-class macro-average F1 score. However, a relatively small improvement of F1 score for very small classes was obtained at the expense of a large amount of false positives, which significantly lowered the overall micro-average F1 score. Ideally, the subject of optimisation would be the micro-average F1 score (since this was the most important measurement in the challenge); however, this would require searching through a vast range of per-class threshold combinations, which, given the amount of classes, is impractical. Instead we opted for simpler and faster, per-class *accuracy*, which proved to be better balanced than the per-class F1 score.

In contrast to SVM, the choice of the subject of optimisation (F1 score vs. accuracy) when choosing the thresholds for the LR models did not affect the performance, which would indicate that the LR models dealt with small classes better than the SVM models. The analysis of the LR thresholds revealed that for the majority of classes the range of prediction probabilities occupied the lower part of the [0, 1] range. Thus, lowering the threshold from the nominal 0.5 boosted recall with an acceptable decrease in precision.

#### Ensemble systems

Having three different systems, we also experimented with two basic types of ensembles: the union and the intersection of the results obtained from the different systems. As shown in Tables 2, 3, and Figure 1, as expected, the intersection systems increased precision, while the union systems increased recall. In particular, the union of m-SVM and b-SVM yielded the best results on the development data and was very competitive on the test data.

### Methods for classifying articles relevant to PPI curation

#### Task analysis

To get a better understanding of the task at hand, we analysed a few randomly chosen positive and negative sample abstracts from the training dataset, in terms of whether the presence of the following attributes in an abstract correlated to its class (i.e., positive or negative): protein names, verbs or nominalised verbs around protein names that signify protein interaction (involving more than one participant), verbs or nominalised verbs near protein names signifying protein modification (one participant), protein name MeSH terms and protein-related biochemical process MeSH terms.

Table 8 shows the results of the analysis of the randomly chosen documents. From this analysis, we speculated that verbs around protein names (A2 in the table) and MeSH terms pertaining to biochemical processes (A5 in the table) could potentially be the most indicative features for distinguishing between positive and negative examples. We hence decided to use contextual words around protein names (to account for the verbs) and MesH terms as features, in addition to bags of words and named entities.

**Table 8:** Prior analysis of 10 sample abstracts

Positive Samples						Negative Samples					
PMID	A1	A2	A3	A4	A5	PMID	A1	A2	A3	A4	A5

17517622	yes	yes	no	yes	yes	19413980	no	no	no	no	no
17586502	yes	no	yes	no	yes	19416831	yes	no	no	yes	no
17666011	yes	yes	no	no	yes	19421224	yes	no	no	no	no
17762861	yes	yes	no	yes	yes	19429605	yes	no	yes	no	no
17942705	yes	yes	yes	yes	no	19435285	yes	no	yes	no	no

The results of both the SVM and LR systems (Tables 9 and 10) confirm the results of our prior analysis. Using context of proteins or MeSH alone gives acceptable performance (micro-averaged F1 score of almost 70%), while using named entities alone produces very poor micro-averaged F1 score. Combining all four features, however, gave the best performance in all metrics except for specificity (where named entities alone performed the best).

**Table 9:** ACT feature knock-out experiments for SVM

Features	F1 score	Specificity	Sensitivity	Accuracy	Matthews Coef	AUC iP/R
B	73.45	93.02	67.95	85.75	64.19	72.44
N	31.75	**98.07**	19.76	75.35	31.50	42.04
C	69.47	93.58	61.58	84.30	60.03	69.98
M	69.07	91.63	63.56	83.49	58.33	68.33

BC	74.93	94.10	68.55	86.69	66.50	73.92
BCM	76.71	94.33	70.86	87.52	68.70	76.00
BNCM	**77.01**	94.48	**71.08**	**87.69**	**69.14**	**76.22**

Results of feature knock-out experiments on the combined ACT training and development datasets (%) with Support Vector Machine (SVM). *B* – bag of words; *N* – named entities; *C* – contextual words surrounding proteins; *M* – MeSH descriptors.

**Table 10:** ACT feature knock-out experiments for LR

Features	F1 score	Specificity	Sensitivity	Accuracy	Matthews Coef	AUC iP/R
B	72.33	91.61	68.28	84.84	62.14	78.97
N	50.05	**94.10**	38.20	77.88	40.75	60.12
C	69.38	89.39	66.91	82.87	57.58	76.30
M	69.61	90.83	65.37	83.44	58.53	75.06

BC	74.57	92.69	70.09	86.13	65.34	80.75
BCM	76.45	93.20	72.17	87.10	67.84	82.67
BNCM	**76.78**	93.49	**72.23**	**87.33**	**68.37**	**82.89**

Results of feature knock-out experiments on the combined ACT training and development datasets for the logistic regression (LR) model (%). *B* – bag of words; *N* – named entities; *C* – contextual words surrounding proteins; *M* – MeSH descriptors.

#### Methods

To approach ACT, we gathered a rich set of features and then used two machine learning paradigms: LR and SVM. Four types of features were extracted: bag of words, named entities (NEs), protein context, and MeSH information. As we only used abstracts to develop our systems, all contextual words were used as bag of words. The types of named entities used were the same as in IMT, namely, proteins, genes, metabolites, organs, drugs, bacteria, diseases, symptoms, diagnostic/therapeutic procedures and phenomena. Since the task was to classify PPI relevant documents, protein names were expected to play an important role. Therefore, we also extracted a range of contextual features from sentences which contain at least one protein name. In more detail, the protein context feature included contextual words without position, and verbs and nouns with position with respect to the protein name.

We employed two types of MeSH information: MeSH terms and MeSH tree structure, where the latter was essentially a tree ID split into one or more substrings. For example, *E05.196.150* would contain 3 MeSH structure features: *E05*, *E05.196*, and *E05.196.150*. The inclusion of this feature alleviated the potential data sparseness problem, caused by the fact that many leaf MeSH IDs occur a very small number of times while non-leaf IDs occur more often, and capturing those could contribute to machine learning models. In addition, we normalised the abbreviations and acronyms to their corresponding long forms [34] in the same documents so that proteins with different names are grounded to a single entity. For example, occurrences of “interleukin 2” and “IL2” in a document would all be converted to “interleukin 2”.

LR and SVM models were then learned from instances of features converted from the training documents. For LR, we used an *L*_2_-regularised model [17,18]. We did similar experiments using SVM*^per f^* and the final models were trained on the combined training and development datasets.

To investigate the contribution from each type of features, we performed feature-knockout experiments. The results with different combinations of features using the SVM system are shown in Table 9.

The most effective feature according to most of the evaluation metrics was the bag of words. Although named entities were the best in terms of specificity, other metrics did not confirm its superiority. The same experiment carried out for the LR system showed a slightly lower overall performance, as shown in Table 10. Despite the fact that the feature contributions were basically the same for both LR and SVM, results obtained when using only named entity features were better for LR than for SVM.

We also compared the results of LR when L1-regularisation was used instead of L2-regularisation. With the features we used, the results with L1-regularisation were slightly worse (0.7 to 1.0% points) than those with L2-regularisations as measured by most evaluation metrics. In particular, it caused more than 10% points drop according to AUC iP/R.

## Competing interests

The authors declaire that they have no competing interests.

## Authors’ contributions

XW led the team, designed and implemented the b-SVM method and the ensemble systems for IMT, and the SVM system for ACT. He also worked on text cleaning, linguistic pre-processing and feature extraction. RR worked on the m-SVM method for IMT, the thresholding strategies, and preliminary versions of the ACT systems. AR created the logistic regression systems for IMT (i.e., m-LR) and ACT. CN created the NER systems and worked on both ACT systems. CR helped on text cleaning and the extraction of MeSH information. RB provided data and error analysis. RN constructed the mapping between MeSH and MI IDs. SA supervised all steps of the work. *All authors* contributed to the preparation of the final manuscript.
